# Assessment of Vitamin Concentrations in Patients with Hashimoto’s Thyroiditis and Their Relationships with Thyroid Function, Biochemical Status, and Anthropometric Parameters—A Preliminary Study

**DOI:** 10.3390/nu16111694

**Published:** 2024-05-29

**Authors:** Aniceta Ada Mikulska-Sauermann, Matylda Resztak, Marta Karaźniewicz-Łada, Dorota Filipowicz, Marek Ruchała, Franciszek K. Główka

**Affiliations:** 1 Department of Physical Pharmacy and Pharmacokinetics, Poznan University of Medical Sciences, Rokietnicka 3, 60-806 Poznan, Poland; mresztak@ump.edu.pl (M.R.); mkaraz@ump.edu.pl (M.K.-Ł.); glowka@ump.edu.pl (F.K.G.); 2 Doctoral School, Poznan University of Medical Sciences, Bukowska 70, 60-812 Poznan, Poland; 3 Department of Endocrinology, Metabolism and Internal Medicine, Poznan University of Medical Sciences, Przybyszewskiego 49, 60-355 Poznan, Poland; dorota.filipowicz123@gmail.com (D.F.); mruchala@ump.edu.pl (M.R.)

**Keywords:** Hashimoto’s thyroiditis, fat-soluble vitamins, water-soluble vitamins, metabolic profile

## Abstract

Hashimoto’s thyroiditis (HT) is the leading cause of hypothyroidism, affecting mainly the female population. Many patients with HT have metabolic disorders and nutritional deficiencies. The aim of this study was to evaluate vitamin D, A, E, B2, and B6 concentrations, thyroid function, metabolic profile, and anthropometric parameters of patients with Hashimoto’s thyroiditis. In 81 female patients with HT (study group), vitamin A and B2 concentrations were significantly lower than in 34 healthy women (control group). No differences were noted in vitamin D, E, and B6 concentrations between groups. Moreover, HT patients had similar anthropometric parameters, lipid profiles, and glucose and insulin concentrations compared to controls. This study showed some relationships between vitamin concentrations and anthropometric or biochemical profiles in HT patients. Among others, in the HT group, the concentration of vitamin D was positively correlated with the level of HDL and negatively correlated with BMI, total fat mass, and insulin level, which influence cardiovascular risk. The results indicate that patients with HT should be routinely tested for vitamin concentrations to prevent nutritional deficiencies. Further studies are also needed on the role of vitamins in the development and progression of HT and the presence of metabolic complications in this population.

## 1. Introduction

Hashimoto’s thyroiditis (HT), also known as chronic lymphocytic thyroiditis, is the most common autoimmune disease and the leading cause of hypothyroidism. The prevalence of HT is a growing trend and mainly affects the female population of any age, with an increased incidence in the middle-aged [1,2,3].

HT pathogenesis is related to the presence of autoreactive lymphocytes infiltrating the thyroid tissue and the production of anti-thyroid peroxidase antibodies (TPOAb) and anti-thyroglobulin antibodies (TgAb). This leads to chronic inflammation, followed by fibrosis and gradual atrophy of the thyroid tissue [1,2,3,4]. In the course of HT, various functional states of the thyroid gland are observed, ranging from normal thyroid function (euthyroidism) through subclinical to overt hypothyroidism [4].

The symptoms of hypothyroidism associated with HT are varied and non-specific (weakness, chronic fatigue, difficulty concentrating, weight changes, and constipation). They concern the cardiovascular, digestive, hematopoietic, and neurological systems, as well as the skin. The symptoms depend on the duration or severity of the disease [2,4].

Genetic, environmental, and existential factors play a role in the pathogenesis of HT. The main genetic factors are polymorphisms in the human leukocyte antigen (HLA) and the cytotoxic 4 T-cell antigen (CTLA-4) gene. The environmental factors include infections, excessive iodine intake, and taking certain medications (e.g., amiodarone or lithium) [2,3,5,6]. Studies also indicate that adequate vitamin D and selenium levels can help to prevent or delay the development of HT [3,7]. This is particularly important during and after pregnancy—a risk factor for autoimmunity—as many women, including those with HT, suffer from selenium deficiencies [8]. In addition, the risk of HT is increased when other autoimmune diseases are present [3].

Treatment of hypothyroidism involves the daily oral administration of the synthetic thyroid hormone levothyroxine (LT4) to maintain normal levels of thyroid-stimulating hormone (TSH) [1,2,4,9]. Moreover, an appropriate supplementation with vitamins and microelements may be a key aspect of the treatment process. However, supplementation guidelines for patients with HT have not yet been developed [10]. Recent recommendations demonstrate that vitamin D supplementation is indicated in people with potential risk factors for vitamin D deficiency or insufficiency, which includes patients with hypothyroidism, common in HT [11,12].

Vitamins are micronutrients that are essential for the proper functioning of many processes in the human body. Recent studies have focused on the role of vitamin D in patients with HT. The special focus is on an association between HT and vitamin D deficiency (assessed by analyzing its main metabolite 25(OH)D) [13]. This is confirmed, among others, by recent systematic reviews and meta-analyses, where significantly lower levels of vitamin D were found in patients with HT compared to healthy people [14,15,16]. However, some studies have shown no differences in vitamin D levels between HT patients and controls [17]. Other studies have explored the role and optimal dosages of vitamin D supplementation in patients with HT [18,19,20]. Most studies have shown that vitamin D supplementation significantly increases 25(OH)D levels and induces changes in TPOAb levels and, in some cases, also TgAb levels in patients with HT. However, no significant relationships were found between the serum vitamin D and TSH, free tetraiodothyronine (fT4), and free triiodothyronine (fT3) concentrations [7,18,20,21]. Some other studies do not indicate a significant effect on thyroid autoimmunity after vitamin D supplementation in patients with HT [22,23].

Patients with HT are characterized by altered antioxidant potential and increased oxidative stress. Many studies have indicated that the level of oxidants is increased and the level of antioxidants is reduced, regardless of the functional state of the thyroid gland in patients with HT compared to healthy people [24,25,26]. Therefore, vitamins such as vitamins A and E, which exhibit antioxidant properties, may play a key role in these patients. Adequate intake of vitamins A, E, C, and group B is also recommended to prevent thyroid diseases, mainly due to the regulation of the pituitary–thyroid axis [27]. Krishnamurthy et al. showed that a deficiency of vitamin B2 significantly affected thyroid function. fT4 levels were significantly lower in those with low vitamin B2 concentrations [28]. Moreover, B vitamins, such as vitamins B2 and B6, are cofactors and coenzymes in many biochemical reactions responsible for proper metabolism [29]. However, vitamin E, A, B2, and B6 concentrations in patients with HT have not been studied so far.

Many patients with HT, even euthyroid, are characterized by excessive body weight and metabolic disorders. Nevertheless, at the same time, in recent years, the incidence of metabolic disorders and obesity has increased [30]. Limited studies assessed the relationship between thyroid autoimmunity in HT patients and the body composition, components of metabolic syndrome, or the risk of cardiovascular disease (CVD). Some studies indicate that HT is associated with obesity and higher cardiovascular risk due to the autoimmune process independent of thyroid function. Other available studies show no such relationship. Therefore, the role of thyroid autoimmunity in metabolic risk remains inconsistent [31,32,33,34,35,36,37].

The aim of this study was to evaluate vitamin D, A, E, B2, and B6 concentrations, thyroid function, metabolic profile, and anthropometric parameters of patients with Hashimoto’s thyroiditis. The associations between those parameters were also considered.

## 2. Materials and Methods

### 2.1. Study Population

This study involved 116 Caucasian women and was conducted in Poland (Poznan; patients were from the area of Greater Poland) between January 2020 and May 2022. Subjects were divided into two groups: 81 female patients with HT (study group) and 34 women without HT (control group). Both groups were recruited simultaneously at the Department of Endocrinology, Metabolism and Internal Medicine at Poznan University of Medical Sciences.

**Figure 1:**
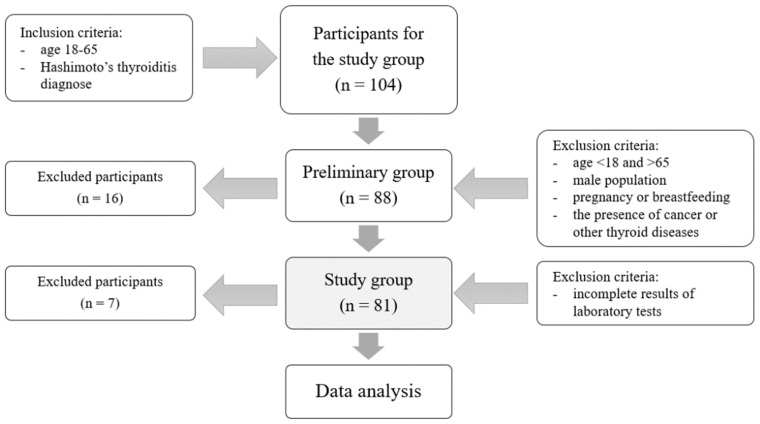
Flow chart of the selection process for the study group.

Inclusion criteria in the study group were diagnosed Hashimoto’s thyroiditis based on biochemical (presence of circulating thyroid autoantibodies) and imaging tests (hypoechogenic inhomogeneous structure of the thyroid gland in ultrasonography) [2,3]. The inclusion criterion for both groups was an age of 18 to 65, and the exclusion criteria were other thyroid diseases, pregnancy, breastfeeding, and a positive history of cancer. Figure 1 presents the selection process of the study group. The control group comprised healthy females without HT (TSH 0.27–2.5 mIU/L, no TPOAb or TgAb, no LT4 use) of a similar age as those in the study group. Moreover, women who had poor health according to physical examination and laboratory analyses were excluded from the control group.

This study was approved by the Local Bioethics Committee at Poznan University of Medical Sciences (no. 873/19 and 201/21). Participation in this study was voluntary. Each participant provided written informed consent after being informed about the project’s purpose and course. This study was carried out in accordance with the Declaration of Helsinki [38].

### 2.2. Anthropometric Parameters

The anthropometric measurements were taken after overnight fasting. During the anthropometric measurements, the patients were dressed only in light underwear, without shoes. Body weight and height were analyzed with an accuracy of 0.1 kg (using a certified weight) and 0.1 cm (stadiometer). Waist circumferences (WC) and hip circumferences (HC) were determined using standard medical instruments. WC was measured at the midway between the costal arch and the upper iliac crest and hip circumference at the level of the greater trochanters. The obtained data were further used to calculate a body mass index (BMI), expressed as weight divided by height squared (kg/m^2^), and waist–hip ratio (WHR), expressed as waist measurement divided by hip measurement. BMI was categorized as underweight (<18.5 kg/m^2^), normal weight (18.5–24.9 kg/m^2^), overweight (25.0–29.9 kg/m^2^), and obese (≥30.0 kg/m^2^) [39].

Additionally, body composition measurements were assessed using bioelectrical impedance analysis, carried out with a TANITA BC-601 device (Tanita Corp., Tokyo, Japan) to estimate total fat mass, total muscle mass, and water content.

### 2.3. Biochemical Parameters

The blood samples of about 7 mL were collected by professional, qualified laboratory personnel from each study participant after overnight fasting (≥12 h after the last meal) and an all-night rest. The serum samples were subjected to biochemical analysis immediately after collection. Biochemical tests included measurements of thyroid and lipid profile. Serum TSH, fT4, fT3, TPOAb, and TgAb concentrations were determined by commercial kits using electrochemiluminescence (ECLIA) by Hitachi and Roche Diagnostics on a Cobas e601 analyzer (Indianapolis, IN, USA). Total cholesterol (TC), high-density lipoprotein (HDL), low-density lipoprotein (LDL), triglycerides (TG), fasting blood glucose (FBG), and insulin were assessed using the enzymatic method with standardized commercial tests (Cobas c, Roche Diagnostic, Mannheim, Germany).

### 2.4. Vitamin Concentrations

Serum and plasma samples were separated by centrifugation of whole blood, and an aliquot was stored at −80 °C until the LC–MS/MS analysis to evaluate vitamin D, A, E, B2, and B6 levels.

Analysis of vitamin D (25-hydroxyvitamin D3), vitamin A (retinol), and vitamin E (α-tocopherol) was performed according to LC-MS/MS method by Karaźniewicz-Łada et al. [40]. The analytes were isolated from plasma samples with liquid–liquid extraction using hexane. Vitamins were separated on the Kinetex F5 analytical column via gradient elution with water and methanol, both with 0.1% (*v*/*v*) formic acid. The analytes were detected on a triple-quadrupole MS with multiple reaction monitoring via electrospray ionization in the positive ion mode.

Vitamin B2 (riboflavin) and B6 (pyridoxal-5′-phosphate) concentration analysis was based on the other LC–MS/MS method. The analytes were separated on Zorbax SB-C8 analytical column. The analytes were isolated from serum by the one-step sample preparation, which involved protein precipitation with trichloroacetic acid. The mobile phase consisted of methanol–water (50:50, *v*/*v*), both containing 0.1% (*v*/*v*) formic acid. Analyte detection was performed on the MS triple-quadrupole as in the previous method [41].

### 2.5. Data Processing and Statistical Analysis

The collected material was coded and entered into the database in Microsoft Excel (Microsoft Corporation, Washington, DC, USA). Statistical analysis of the results was conducted using Statistica 13 software (StatSoft, Tulsa, OK, USA). The normality of the data distribution was evaluated with the Shapiro–Wilk test. The results are presented as means ± standard deviations (SD). The results were analyzed statistically, using elements of descriptive statistics and statistical procedures, such as correlation analysis (Pearson’s for parametric distributions and Spearman’s for nonparametric distributions). Student‘s *t*-test was used to compare variables in normal distribution, Mann–Whitney U test was applied for non-normal data distributions, and categorical variables were compared using the chi-square test. The multivariate regression analysis was applied to evaluate the co-influence of several variables on biochemical and metabolic parameters. *p* < 0.05 were considered statistically significant.

## 3. Results

One hundred sixteen women aged 39.6 ± 13.9 years were enrolled in this study. Nutritional status was assessed based on the World Health Organization (WHO) criteria for BMI [39]. The mean BMI of the entire study population was 26.09 ± 5.23 kg/m^2^ and was not significantly different between the groups (*p* > 0.05). Most participants from the study group had normal body weight. Additionally, 44.4% of patients and more than half of the controls were overweight or obese. Table 1 demonstrates the nutritional status of the studied groups based on the BMI index.

The results of the anthropometric analysis showed statistically significant differences in WHR. The individuals with HT were characterized by slightly lower BMI, total fat mass, WC, and HC than those without HT, but the differences were not statistically significant (Table 2). In addition, no differences were noted in lipid profile, FBG, and insulin concentrations between patients with HT and control subjects. Due to the presence of TPOAb and TgAb in patients with HT and their absence in people from the control group, there was a statistically significant difference in these parameters between the analyzed groups. The characteristics of the populations are summarized in Table 2.

As shown in Table 3, statistically significant differences between the groups occurred in vitamin A and vitamin B2 concentrations. In the present study, the HT group had statistically lower vitamin A (6.24% lower; *p* = 0.003) and vitamin B2 concentrations (41.03% lower; *p* = 0.003) as compared with the control group. Vitamin D, E, and B6 concentrations were slightly lower in HT patients than in healthy controls but without a statistically significant difference (*p* > 0.05). Moreover, in the study group, 58.02% had vitamin D deficiency (<20 ng/mL), 22.22% had suboptimal status (20–30 ng/mL), and 18.52% had an optimal concentration (30–50 ng/mL), while in the control group, there were 52.94%, 32.35%, and 11.76%, respectively. The results of the vitamin concentrations in patients with HT and healthy controls are presented in Table 3.

In the entire studied population, the concentration of vitamin D was significantly positively correlated with HDL and negatively with total fat mass and insulin level (Table 4). In addition, a negative association was found between vitamin B2 concentration and TSH level (R = −0.293; *p* < 0.05). In the entire population, the concentration of vitamin A was significantly positively correlated with anthropometric markers, like BMI, WHR, and total fat mass, and with the metabolic components, such as TG and insulin (Table 4). Furthermore, as shown in Table 4, in the entire research group, vitamin E concentration correlated positively with BMI, WHR, total fat mass, levels of TC, LDL, TG, and FBG. There was no relationship between the level of TPOAb and the concentration of vitamins, lipids, or thyroid profile.

In the HT group, a positive correlation existed between vitamin D concentration and HDL level. Furthermore, a negative association was noted between vitamin D concentration and BMI, total fat mass, and insulin level (Table 5). In the study group, vitamin E level correlated positively with BMI, WHR, TC, LDL, TG, and FBG (Table 5). Similar positive relationships were observed between vitamin A concentration and BMI, WHR, total fat mass, TG, and insulin level. Moreover, in HT patients, vitamin B2 concentration negatively correlated with TSH (R = −0.315; *p* < 0.001). In the case of vitamin B6, there was only a negative correlation with total fat mass (R = −0.228; *p* < 0.001).

In the control group, the concentration of vitamin B2 significantly positively correlated with fT4 level (R = 0.385; *p* < 0.05). Our findings also indicated a positive relationship between vitamin E concentration and levels of TC (R = 0.456; *p* < 0.05), LDL (R = 0.510; *p* < 0.05), and TG (R = 0.531; *p* < 0.05). Furthermore, in healthy controls, the concentration of vitamin B6 correlated negatively with the TPOAb level (R = −0.362; *p* < 0.05).

In the study group, the multivariate regression analysis showed the significant effect of BMI and HDL levels on vitamin D concentration (Table 6). Moreover, TC and TG co-influenced vitamin E levels. For vitamin A, only insulin concentration proved to influence the vitamin A body status, and a combination of other factors did not have such an effect.

In the entire studied population, the multivariate regression analysis showed the significant effect of BMI, LDL and total fat mass on vitamin D concentration. Moreover, total fat mass and insulin level were determinants of vitamin A concentrations while TG and TC co-influenced vitamin E levels (Table 7).

## 4. Discussion

Our study documented similar anthropometric parameters, such as BMI, total fat mass, WC, and HC, in the HT population and healthy controls. The results of the anthropometric analysis showed only statistically significantly lower WHR in HT patients compared to the control group. Moreover, in our study, there were no differences in lipid profile, FBG, and insulin concentrations between studied populations. Some other studies also indicated that HT patients have similar anthropometric and metabolic profiles compared to people without autoimmunity [34,36,37]. In a cross-sectional study with 99 HT patients and 202 controls, the anthropometric and metabolic parameters, including BMI, total fat mass, insulin, and TG levels, were similar in both groups. The prevalence of metabolic syndrome was also comparable in HT and control groups [36]. Moreover, Liu et al. found that patients with autoimmune thyroiditis and healthy controls did not significantly differ in BMI, TC, LDL, HDL, TG, and FBG levels [34]. In another study conducted by Pan et al. with 1105 participants (mainly women), there were no differences in BMI, the prevalence of diabetes, hypertriglyceridemia, and hypercholesterolemia between participants with and without thyroid autoantibodies [37]. On the other hand, the systematic review and meta-analysis by Song et al., which included 22 studies, demonstrated that obesity is significantly associated with HT and high levels of TPOAb [33]. Furthermore, a study by Poplawska-Kita et al. has shown significantly higher body weight, BMI, WHR, and total fat mass in 53 patients with HT than in 28 healthy participants [35]. The study with 50 patients with subclinical hypothyroidism due to HT and 50 controls demonstrated that LDL level was higher and HDL level lower in the HT group compared with the control group [42]. In the cross-sectional study, a group of TPOAb/TgAb-positive women had significantly increased BMI, WC, TC, and LDL cholesterol compared to controls. This study also indicated that obesity and high LDL cholesterol are associated with increased positive thyroid autoantibody levels [43].

According to our knowledge, this is the first study to establish vitamin B2, B6, A, and E status in the HT population. The present study documented statistically significant lower vitamin B2 concentration in HT patients compared to the control group. The concentration of vitamin B2 in HT patients was similar to those reported by Geng et al., where the mean concentrations in patients suffering from vitamin-related diseases were 5.86 ng/mL [44]. Our findings revealed that in the whole studied population, including patients with HT, elevated vitamin B2 concentrations were associated with decreased TSH levels, indicating a positive effect on thyroid function. In addition, in the control group, the association between the increased concentration of vitamin B2 and the increase in the level of fT4 was observed. A study by Krishnamurthy et al. also observed that vitamin B2 was significant for normal thyroid functioning [28]. However, the mechanism of how this vitamin affects the thyroid gland has not yet been elucidated. We did not show any relationship between vitamin B2 concentrations and anthropometric or metabolic parameters.

In our study, no differences in vitamin B6 concentrations were observed in the studied populations. In an abovementioned study conducted by Geng et al., vitamin B6 concentration was lower than in both our studied groups [44]. Moreover, we observed associations between increased vitamin B6 concentration and a decrease in total fat mass levels in HT patients and reduced TPOAb levels in the control group. As already mentioned in Section 1, vitamin B6 plays an important role in energy metabolism. Its deficiency may stimulate fat production and be involved in the development of obesity and other metabolic diseases [29]. Higher levels of vitamin B6 may improve fat tissue distribution and reduce total fat mass in HT patients. Therefore, it may positively influence metabolic parameters.

The current findings revealed that patients with HT had significantly lower concentrations of vitamin A compared to the control group. Results from another study by Pang et al. showed similar values of retinol concentrations in healthy controls as in our study (408.84 ng/mL) [45]. Vitamin A has antioxidant and anti-inflammatory properties; therefore, we assume that its reduced concentration in patients with HT may have an adverse effect on thyroid autoimmunity and the metabolic profile. In this study, we have shown in the study group a relationship between elevated vitamin A concentration and increased BMI, WHR, total fat mass, and levels of TG and insulin. Moreover, we indicated that insulin level was a determinant of vitamin A concentration. Qorbani et al. also found that retinol concentration was positively associated with metabolic syndrome components, such as obesity, low HDL, and high FBG levels. They also indicated that vitamin A level and its association with metabolic response is influenced by some genetic variations [46]. We did not observe any significant correlation between vitamin A and thyroid function parameters. The same results were obtained by Krishnamurthy et al. [28].

The results of our study indicate that patients with HT and the healthy group showed similar values of vitamin E. This study also demonstrated a relationship between the concentration of vitamin E and anthropometric parameters and lipid profile in HT patients, indicating that increased concentration of vitamin E is associated with elevated BMI, WHR, levels of TC, LDL, TG, and FBG. Moreover, TC and TG levels co-influenced vitamin E concentration. Similar relationships were found in the entire study population. No data on the correlation between vitamin B2, B6, A, or E and anthropometric or biochemical parameters are available in patients with HT to compare our results. Our study is the first analysis of these associations in this population.

25-hydroxyvitamin D ranges for vitamin D deficiency is <20 ng/mL, suboptimal status is between 20 and 30 ng/mL, and optimal concentration is 30–50 ng/mL [11,47]. In both studied groups, more than half of the participants had vitamin D deficiency, and less than 20% had optimal concentration. The difference in vitamin D concentrations was not statistically significant between groups. Similar results were demonstrated by Botelho et al. Vitamin D concentrations were similar in 88 patients with HT and 71 healthy controls [17]. Another study also showed no significant differences in vitamin D levels between 461 HT patients and 176 healthy controls. However, the results of the study indicated a decrease in vitamin D levels with the progression of the disease [48]. Different results from ours were obtained by Maciejewski et al. They found that vitamin D concentration was significantly lower in 62 HT patients in comparison to 32 healthy controls (8.04 ng/mL vs. 12.12 ng/mL) [49]. Moreover, the epidemiological study by Fang et al. has demonstrated that vitamin D deficiency was associated with TPOAb positivity and that vitamin D deficiency seems to be involved in the pathological mechanism underlying HT [50]. Recent studies by Ucan et al. with 75 HT patients and 43 healthy individuals indicated that thyroid autoantibodies were significantly decreased by vitamin D treatment in patients with HT. Moreover, HDL cholesterol levels increased after treatment of vitamin D deficiency in the HT group. Therefore, their study has suggested that vitamin D supplementation may slow down the development of hypothyroidism and reduce the cardiovascular risk in these patients [19]. Our study confirmed that in the HT group, the concentration of vitamin D was positively correlated with the level of HDL. The multivariate regression analysis indicated the significant effect of BMI and HDL levels on concentrations of vitamin D. We have also demonstrated a negative correlation between vitamin D concentrations and BMI, total fat mass, and insulin level, which influence cardiovascular risk. Recent studies have shown an improvement in the metabolic profile of people receiving vitamin D supplementation. However, the mechanism by which metabolic risk is reduced is not fully elucidated [51]. This may be due to the influence of vitamin D on adipose tissue parameters and adipocyte biology, which has been demonstrated in vitro studies [52]. Additionally, proper vitamin D supply may have a positive impact on the functioning of the immune system and redox homeostasis, demonstrating antioxidant, anti-inflammatory, and immunomodulatory effects.

### Limitations and Strength of the Study

The research model presented here has some limitations. The first is the small number of individuals in the study and control groups. Moreover, this study only included female Caucasians. Expanding studied groups and including more diverse populations (male patients and different races) is worth considering. Another limitation is that the research included patients with HT with various thyroid functional states ranging from subclinical euthyroid to overt hypothyroidism. Another limitation is the lack of division between people with and without supplementation of the analyzed vitamins due to the research group being too small. People from both the study and control groups most often supplemented vitamin D and magnesium during this study. Vitamin D was supplemented by 39.36% of the study group and 35.29% of the control group, and magnesium was supplemented by 23.40% and 14.71%, respectively. We do not have information about previous supplementations.

The key strength of this study included the assessment of vitamin D, A, E, B2, and B6 concentrations in patients with Hashimoto’s thyroiditis. To the best of our knowledge, this is the first study to establish vitamin concentrations and an in-depth, comprehensive analysis of their relationships with thyroid function, biochemical status, and anthropometric parameters.

## 5. Conclusions

Our results indicate that patients with Hashimoto’s thyroiditis showed reduced vitamin B2 and vitamin A levels compared to healthy controls. The concentrations of vitamins D, E, and B6 did not differ between the study and control groups. However, more than half of patients with HT showed a vitamin D deficiency. Moreover, the HT group had similar anthropometric parameters, lipid profile, glucose, and insulin concentrations compared to controls without autoimmunity. The present study demonstrated that in HT patients, the concentration of vitamin D was positively correlated with the level of HDL and negatively with BMI, total fat mass, and insulin level, which influence cardiovascular risk. We have also shown that increased vitamin B6 concentration was associated with a decrease in total fat mass levels in the study group. Furthermore, in this group, there was a positive correlation between vitamin A concentration and BMI, WHR, total fat mass, and levels of TG and insulin. In patients with HT, positive correlations between the concentration of vitamin E and particular components of the metabolic syndrome were also observed, which indicates a significant role of this vitamin in the course of HT. Currently, no data on the correlation between the analyzed vitamins and anthropometric or biochemical parameters are available for patients with HT to compare our results. Our study is the first analysis of these associations in this population. In conclusion, the study results indicate that patients with HT should be routinely tested for vitamin concentrations to prevent nutritional deficiencies. Further studies are needed to assess the effects of vitamins on thyroid function, autoimmunity, and metabolic complications in the HT population. In the future, guidelines for vitamin supplementation in patients with HT should be established based on evidence-based medicine (EBM), especially in HT patients with concomitant metabolic disorders.

## Figures and Tables

**Table 1:** Nutritional status of studied groups.

BMI Value	Nutritional Status	Study Group (n = 81)	Control Group (n = 34)	*p*-Value
<18.5	Underweight	3 (3.7)	0 (0.0)	NS
18.5–24.9	Normal weight	42 (51.9)	14 (41.2)	NS
25–29.9	Overweight	19 (23.4)	9 (26.5)	NS
≥30	Obese	17 (21.0)	11 (32.3)	NS

The parameter values are presented as means (±SD); n (%)—number of individuals; *p*—statistical significance level; BMI—body mass index; NS—difference not statistically significant.

**Table 2:** Characteristics of the studied populations.

	Study Group (n = 81)	Control Group (n = 34)	*p*-Value
**Age [years]**	40.79 ± 14.25	36.91 ± 12.75	NS
**BMI [kg/m^2^]**	25.60 ± 5.26	27.26 ± 5.04	NS
**WC [cm]**	82.85 ± 14.10	84.50 ± 10.54	NS
**HC [cm]**	102.85 ± 10.10	101.05 ± 6.87	NS
**WHR**	0.80 ± 0.08	0.83 ±0.07	*p* = 0.04
**Total fat mass [%]**	33.17 ± 8.55	36.26 ± 7.17	NS
**TSH [mIU/L]**	2.70 ± 7.44	1.56 ± 0.67	NS
**TPOAb [IU/mL]**	181.00 ± 186.16	10.94 ± 4.38	*p* = 1 × 10^−12^
**TgAb [IU/mL]**	258.42 ± 468.26	13.82 ± 2.52	*p* = 2.2 × 10^−13^
**fT3 [pmol/L]**	4.96 ± 2.88	4.53 ± 0.49	NS
**fT4 [pmol/L]**	17.37 ± 6.33	14.99 ± 2.00	*p* = 0.003
**TC [mg/dL]**	191.51 ± 46.02	201.94 ± 51.59	NS
**HDL [mg/dL]**	63.52 ± 16.79	59.24 ± 13.87	NS
**LDL [mg/dL]**	117.90 ± 42.11	125.38 ± 43.38	NS
**TG [mg/dL]**	128.01 ± 91.84	135.06 ± 86.28	NS
**FBG [mg/dL]**	102.59 ± 26.62	95.21 ± 11.02	NS
**Insulin [mU/mL]**	12.14 ± 8.45	14.07 ± 9.60	NS

The parameter values are presented as means (±SD); n—number of individuals; *p*—statistical significance level according to the Mann–Whitney U-test for study vs. control groups in the case of nonparametric distributions or the T-test for parametric data; NS—difference not statistically significant; BMI—body mass index; WC—waist circumference; HC—hip circumference; WHR—waist-to-hip ratio; TSH—thyroid stimulating hormone; TPOAb—anti-thyroid peroxidase antibodies; TgAb—anti-thyroglobulin antibodies; fT3—free triiodothyronine; fT4—free thyroxine; TC—total cholesterol; HDL—high-density lipoprotein; LDL—low-density lipoprotein; TG—triglycerides; FBG—fasting blood glucose.

**Table 3:** Concentrations of vitamins in the study and control groups.

Parameter [Unit]	Study Group (n = 81)	Control Group (n = 34)	*p*-Value
**Vitamin D** [ng/mL]	19.58 ± 11.20	21.78 ± 11.18	NS
**Vitamin A** [ng/mL]	395.88 ± 385.56	422.21 ± 134.98	*p* = 0.003
**Vitamin E** [μg/mL]	16.23 ± 6.36	16.64 ± 6.10	NS
**Vitamin B2** [ng/mL]	7.13 ± 7.59	12.09 ± 9.62	*p* = 0.003
**Vitamin B6** [ng/mL]	30.89 ± 19.49	37.29 ± 23.02	NS

The parameter values are presented as means (±SD); n—number of individuals; NS—difference not statistically significant; *p*—level of statistical significance for study group vs. control group according to Student’s *t*-test or Mann–Whitney U test (for, respectively, normal or non-normal data distributions).

**Table 4:** Indices of correlations and levels of statistical significance in cases of the relationship between vitamin A and E and selected anthropometric and biochemical parameters in entire studied population.

Parameter	Vitamin D	Vitamin A	Vitamin E
**BMI**	NS	R = 0.353 *p* < 0.05	R = 0.232 *p* < 0.05
**WHR**	NS	R = 0.347 *p* < 0.05	R = 0.408 *p* < 0.001
**Total fat mass**	R = −0.208 *p* < 0.05	R = 0.258 *p* < 0.05	R = 0.210 *p* < 0.05
**TC**	NS	NS	R = 0.613 *p* < 0.000001
**TG**	NS	R = 0.330 *p* < 0.05	R = 0.541 *p* < 0.000001
**LDL**	NS	NS	R = 0.565 *p* < 0.000001
**HDL**	R = 0.199 *p* < 0.05	NS	NS
**Insulin**	R = −0.268 *p* < 0.05	R = 0.328 *p* < 0.05	NS
**FBG**	NS	NS	R = 0.276 *p* < 0.05

Parameters are shown as R—coefficient of Pearson or Spearman (for, respectively, normal or non-normal data distributions); *p*—level of statistical significance; NS—statistically non-significant difference; BMI—body mass index; WHR—waist-to-hip ratio; TC—total cholesterol; TG—triglycerides; LDL—low-density lipoprotein; HDL—high-density lipoprotein; FBG—fasting blood glucose.

**Table 5:** Indices of correlations and levels of statistical significance in cases of analyses of the relationship between vitamins and selected anthropometric and biochemical parameters in the study group.

Parameter	Vitamin D	Vitamin A	Vitamin E
**BMI**	R = −0.224 *p* < 0.001	R = 0.391 *p* < 0.001	R = 0.261 *p* < 0.001
**WHR**	NS	R = 0.370 *p* < 0.001	R = 0.416 *p* < 0.001
**Total fat mass**	R = −0.278 *p* < 0.001	R = 0.261 *p* < 0.001	NS
**TC**	NS	NS	R = 0.694 *p* < 0.000001
**TG**	NS	R = 0.395 *p* < 0.001	R = 0.563 *p* < 0.000001
**LDL**	NS	NS	R = 0.598 *p* < 0.000001
**HDL**	R = 0.257 *p* < 0.001	NS	NS
**Insulin**	R = −0.421 *p* < 0.001	R = 0.508 *p* < 0.001	NS
**FBG**	NS	NS	R = 0.271 *p* < 0.001

Parameters are shown as R—coefficient of Pearson or Spearman (for, respectively, normal or non-normal data distributions); *p*—level of statistical significance; NS—statistically non-significant difference; BMI—body mass index; WHR—waist-to-hip ratio; TC—total cholesterol; TG—triglycerides; LDL—low-density lipoprotein; HDL—high-density lipoprotein; FBG—fasting blood glucose.

**Table 6:** Results of multivariate regression analysis for vitamin A, D, and E concentrations in the study group.

Variables	B Coefficient		*p*-Value
	Value	Standard Error	
**Vitamin D**			
BMI	0.711	0.329	0.0408
HDL	0.508	0.186	0.0114
*p* value of the model = 0.035, R = 0.573			
**Vitamin E**			
TC	0.825	0.280	0.0054
TG	0.565	0.110	<0.0001
*p* value of the model < 0.0001, R = 0.877			

R—correlation coefficient for the model; *p*—level of statistical significance; HDL—high-density lipoprotein; BMI—body mass index; TC—total cholesterol; TG—triglycerides.

**Table 7:** Results of multivariate regression analysis for vitamin A, D and E concentrations in the entire studied population.

Variables	B Coefficient		*p*-Value
	Value	Standard Error	
**Vitamin D**			
BMI	−1.22	0.435	0.0077
HDL	1.43	0.428	0.0018
Total fat mass	−0.31	0.139	0.0302
*p* value of the model = 0.0015, R = 0.568			
**Vitamin A**			
Total fat mass	0.264	0.129	0.0450
Insulin	0.0369	0.129	0.0058
*p* value of the model < 0.0001, R = 0.548			
**Vitamin E**			
TG	0.524	0.0679	<0.0001
TC	0.394	0.0679	<0.0001
*p* value of the model < 0.0001, R = 0.765			

R—correlation coefficient for the model; *p*—level of statistical significance; BMI—Body Mass Index; LDL—low-density lipoprotein; TC—total cholesterol; TG—triglycerides.

## Data Availability

The data presented in this study are available on request from the corresponding author. The data are not publicly available due to privacy restrictions.
